# DPDR-CPI, a server that predicts Drug Positioning and Drug Repositioning via Chemical-Protein Interactome

**DOI:** 10.1038/srep35996

**Published:** 2016-11-02

**Authors:** Heng Luo, Ping Zhang, Xi Hang Cao, Dizheng Du, Hao Ye, Hui Huang, Can Li, Shengying Qin, Chunling Wan, Leming Shi, Lin He, Lun Yang

**Affiliations:** 1 Bio-X Institutes, Shanghai Jiao Tong University, Shanghai 200030, China; 2 Center for Computational Health, IBM T.J. Watson Research Center, Yorktown Heights, NY 10598, USA; 3 Center for Data Analytics and Biomedical Informatics, Temple University, Philadelphia, PA 19122, USA; 4 Collaborative Innovation Center for Genetics and Development, State Key Laboratory of Genetic Engineering and MOE Key Laboratory of Contemporary Anthropology, School of Life Sciences, Fudan University, Shanghai 200438, China

## Abstract

The cost of developing a new drug has increased sharply over the past years. To ensure a reasonable return-on-investment, it is useful for drug discovery researchers in both industry and academia to identify all the possible indications for early pipeline molecules. For the first time, we propose the term computational “drug candidate positioning” or “drug positioning”, to describe the above process. It is distinct from drug repositioning, which identifies new uses for existing drugs and maximizes their value. Since many therapeutic effects are mediated by unexpected drug-protein interactions, it is reasonable to analyze the chemical-protein interactome (CPI) profiles to predict indications. Here we introduce the server DPDR-CPI, which can make real-time predictions based only on the structure of the small molecule. When a user submits a molecule, the server will dock it across 611 human proteins, generating a CPI profile of features that can be used for predictions. It can suggest the likelihood of relevance of the input molecule towards ~1,000 human diseases with top predictions listed. DPDR-CPI achieved an overall AUROC of 0.78 during 10-fold cross-validations and AUROC of 0.76 for the independent validation. The server is freely accessible via http://cpi.bio-x.cn/dpdr/.

The cost of developing a new drug increased from $0.8 billion in 2003 to $2.6 billion in 2014^1^. It was estimated that only one drug compound was approved for market use after screening, selection and trials from a large number of compounds within 10–17 years^2^,^3^. The research and development (R&D) costs of new drugs are increasing while the number of annual approved new drugs has not changed much^4^. Therefore, it is important for drug developers in industry or academia to identify all possible indications for their pipeline molecules, i.e., positioning the molecule towards the best possible indications as early as possible. Even if there is no clinical or animal data available for the molecule, which is usually the case at early stages of the pipeline, potential indications should be identified. Here, for the first time, we propose this indication prioritization process as “drug candidate positioning”, or “drug positioning”, which differentiates with “drug repositioning” and could be one of the essential steps in the future R&D strategy. On the other hand, drug repositioning, i.e., identifying new uses for existing drugs^3^, also could maximize the market value of the existing drugs^5^. For both positioning and repositioning, the process of computational indication prediction is essential.

Many computational methods have been developed for drug repositioning, including structure-based prediction^6^, side-effect-based approach^7^,^8^, networks^9^,^10^,^11^, gene expression analysis^12^,^13^,^14^,^15^,^16^ and text mining^17^. Some studies combined various data types to get improved prediction performance^18^,^19^. Servers that utilize descriptors^20^,^21^, gene expressions^13^,^22^ and multiple data types^11^ were developed. Most of the above methods require data and knowledge that have already been generated, such as the associated drug targets, drug labels, gene expression profiles and side-effects, many of which are only applicable to the late-stage or marketed drugs but not to early pipeline molecules. Therefore, they are not available to support drug candidate positioning. During our previous studies, we address this issue by constructing the *in silico* chemical-protein interactome (CPI)^6^,^23^,^24^,^25^,^26^,^27^, based on which the DRAR-CPI was developed^6^. The server requires the user submission of a molecular structure via the web interface, and then a CPI profile will be constructed for indication prediction. The CPI profile will be compared against the profiles of our library drugs and potential indications will be suggested based on profile similarities. It has helped different groups of researchers to identify putative targets and potential indications for their molecules^28^,^29^,^30^,^31^. However, the server was developed five years ago and it has two major limitations: (a) the number of predicted indications are limited and biased because of the limited drug library in our server and (b) the indication prediction is based on an unsupervised method, which does not utilize a training process to optimize the prediction for each indication. Therefore, we introduce an upgraded version of the server, DPDR-CPI, to predict drug candidate positioning and drug repositioning via CPI. It can accept a small molecule in major formats, including MOL, MOL2, PDB, SDF and SMILES, and predict its potential indications across 963 diseases using machine learning models. The performances were validated using a blinded independent validation–the model was trained at one institution and validated another institution. It achieved an area under the receiver operating characteristic curve (AUROC) of 0.78 during 10-fold cross-validations. The server will also suggest putative targets and their docking conformations based on a faster and more accurate docking program so that the users can explore the rationale of the predicted indications^32^.

## Results and Discussion

### Model evaluation

The training set and the independent validation set both contain 628 drugs and 638 ICD-9 disease indications belonging to 328 ICD-9 disease families (Supplementary Tables S1 and S2). For the 10-fold cross-validations of the training set under global metrics, the models obtained an AUROC of 0.752 for the 638 ICD-9 disease indications and 0.760 for the 328 ICD-9 disease families. The server-side models were trained using the combination of both the training set and the independent validation set (called entire dataset). They reached an AUROC of 0.782 for the 638 ICD-9 disease indications and 0.783 for the 328 ICD-9 disease families. Other measurements, including accuracy, precision, sensitivity, specificity and area under the precision-recall curve (AUPR), are shown in Table 1.

**Figure 1:**
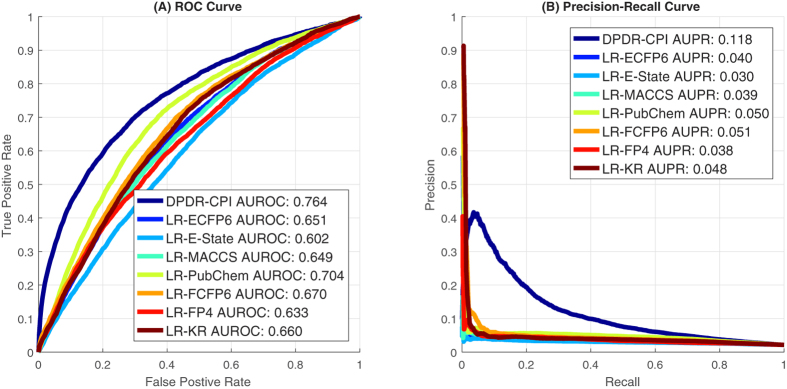
Under global metric, (**A**) the ROC curve comparison and (**B**) the precision-recall curve comparison for different prediction methods for 638 ICD-9 disease indications on the independent validation data.

For the independent validation, we compared two types of prediction methods: (1) logistic regressions based on E-state, Extended Connectivity Fingerprint (ECFP)-6, Functional-Class Fingerprints (FCFP)-6, FP4, Klekota-Roth method, MACCS and PubChem structural descriptors (called LR-E-state, LR-ECFP6, LR-FCFP6, LR-FP4, LR-KR, LR-MACCS and LR-PubChem, respectively)^33^, and (2) DPDR-CPI proposed in this paper that analyzes CPI profiles to predict indications. For the 638 ICD-9 disease indications as endpoints, the comparisons of receiver operating characteristic (ROC) curves and precision-recall curves under global metrics are shown in Fig. 1. All evaluation measurements including global, drug-centric and disease-centric metrics are summarized in Table 2. We see the DPDR-CPI obtained the best overall performance with an AUROC of 0.764 during the independent validation.

Likewise, we used 328 ICD-9 disease families as endpoints and compared the structural descriptor-based methods and DPDR-CPI. The ROC and precision-recall curves are shown in Supplementary Figure S1 and evaluation measurements are attached in Supplementary Table S3. From either ICD-9 diseases or ICD-9 disease families, the independent validation showed that our CPI-based method generally outperformed structural descriptor-based methods. DPDR-CPI achieved a reasonably good overall performance and can be utilized for drug candidate positioning and repositioning purposes.

The CPI is an *in silico* atomistic prediction of drug-protein binding data. Though some studies utilized experimental drug-protein binding data to predict drug indications and demonstrated good prediction performances^18^,^19^,^34^, such information is limited for new or pipeline drug candidates. Though our CPI may not be as accurate as the experimental binding data, it has the advantage to make predictions for new or pipeline drug candidates. Since obtaining the wet-lab binding data can be both costly and time-consuming, we believe our CPI provides a fast, low-cost and useful solution for drug candidate positioning.

Another advantage of our CPI approach is the consideration of potential off-target binding effects, which are important to the discovery of new indications. The 611 targets in our library consist of both pharmacokinetic (PK) and pharmacodynamic (PD) proteins serving as a reasonable distribution of off-targets. The features provided by off-target binding effects can be used to identify drug indications even if the on-target does not exist in the library. For example, Rolapitant is a neurokinin-1 (NK-1) receptor antagonist that can treat vomiting. Even though its target NK-1 is not included in our library, we submitted the molecule to our DPDR-CPI server and found its indication ranked to top second with a high confidence value of 0.85.

Since drugs in the independent validation set may have similar structures to some of the drugs used in the training set, to reduce such impact, we removed the drugs from the independent validation set which have a Tanimoto similarity >0.7^18^ towards any drug in the training set. The new results of independent validation are shown in Supplementary Tables S4 and S5. We see that after removing the similar drugs, the AUROC of DPDR-CPI slightly dropped by 0.02~0.03, indicating the performance of our method is not mainly contributed by structural similar drugs.

### Case study 1: drug candidate positioning for parogrelil

It is important to make early decisions of the indication prioritization for the pipeline molecules, so that the developers could choose the best indications with unmet needs, clinical developability and return on investment. Here we found an investigational molecule, “NM-702”, originally developed for peripheral vascular disease^35^ (http://www.drugbank.ca/drugs/DB05505), and submitted it the DPDR-CPI server. The server successfully picked up this indication (Table 3) as the third rated one and all the top four predictions were relevant to the same disease category (cardiovascular diseases). Among these four top predictions, we believe that the second prediction, cerebral arterial occlusion, is a highly unmet need and should be considered. Acute stroke is caused by cerebral arterial occlusion and can lead to brain infarction^36^. Stroke is the fifth most common cause of death and the most frequent causes of disability in the US^37^. Therefore, by using the DPDR-CPI server, the drug developer could have positioned this drug candidate into the second indication and compared efficacy for both indications in the respective animal models. We believe the server provides drug developers an opportunity to choose the most promising indication for further development, such as deciding whether to pursue it for a higher unmet need (cerebral artery occlusion) or continuing its original designated indication, along with the atomistic docking model to help make sense of the additional targets.

From the case study, we see that the DPDR-CPI server can identify the best indications for a compound based only on its molecular structure, which is very important to the pharmaceutical industry since it supports a rapid high throughput approach. Though our work is based on the *in silico* docking approach, which has been extensively used for virtual screening and target identification in the past decades, the purposes of this work include drug candidate positioning as an important application.

### Case study 2: drug repositioning for rosiglitazone

Rosiglitazone is an anti-diabetic drug which has been on the market for years. We would like to know whether our server is able to expand its indications for possible new uses. We submitted its structure to the server and found our server successfully identified its original indications, hypoglycemia and diabetes mellitus, as the top two predictions (Table 4). Some other reported new uses, such as disorders of fatty acid oxidation^38^ and Alzheimer’s disease^39^, are also prioritized by the server. Among the top predictions, retinal disorders and glaucoma are also listed. It is reported that rosiglitazone is a potential neuroprotectant for retinal cells and may increase the retinal cell survival^40^. It may also delay the onset of proliferative diabetic retinopathy^41^. In addition, the drug was found useful after glaucoma filtration surgery for anti-fibrotic activity^42^. Therefore, in concordance with the literature reports, the atomistic based prediction results suggested that it is possible to expand rosiglitazone for eye disease treatments.

We also look at the binding target predictions for rosiglitazone and found monoamine oxidase A (MAO-A) is ranked in the top three. It was reported that rosiglitazone is an inhibitor for MAO-A^43^, a drug target for neuroprotective therapy^44^. Such prediction provides possible biologic clues for rosiglitazone’s neuroprotective effects towards retinal cells, and may help to discover its potential uses and mechanisms for treating eye diseases.

## Conclusion

The DPDR-CPI server is able to produce indication predictions for a user molecule towards ~1,000 human diseases, providing suggestions for drug candidate positioning and drug repositioning. It has the potential to improve the drug development pipeline in terms of indication prioritization even for molecules in the early R&D stage.

## Methods

### Preparation of the training set

We included 2,515 drug molecules, 611 ligand-bindable target structures and their CPI from our previous study^24^. The 2,515 molecules were collected from DrugBank^45^ and STITCH^46^, of which 85% are FDA-approved drugs. The 611 target structures contains 239 PK proteins and 372 PD proteins collected from Protein Data Bank (PDB)^47^ and PDBBind^48^. Though the targets were harvested from a project for drug-drug interaction prediction, we still believe they can serve as potential off-target binding features for drug indication prediction. The *in silico* interactome of these 2,515 molecules across 611 targets was generated using AutoDock Vina^32^.

We chose MEDication Indication resource (MEDI)^49^ as a gold standard for drug indications since it contains the largest number of indications (4,352 diseases) among the existing drug-indication databases^50^ and it uses International Classification of Disease (ICD)-9 codes (2014 version) to represent diseases. We mapped the 3,112 drugs from MEDI to DrugBank using DrugBank synonym rules, and identified 1,256 common drugs that exist both in MEDI and our CPI (Supplementary Table S1). The docking scores of 1,256 common drugs against the 611 targets were used as features for our machine learning models, and the disease indications are considered as endpoints.

We filtered the endpoints according to the following criteria: (a) we removed the endpoints containing ICD-9 codes from 780 to 999 since they are related to symptoms, injuries or poisoning which are less of interest; (b) we removed the endpoints that can be treated by less than five drugs due to the fact that the positive samples are too few in those cases. Afterwards, we got 963 ICD-9 disease indications which belong to 424 ICD-9 families (Supplementary Table S2). For each drug-indication pair, if the drug is reported to treat the indication in MEDI, it is labeled as “1” (positive), otherwise, “0” (negative). Finally, the dataset was converted to a matrix containing 1,256 drugs as rows and 611 target-binding features as predictor variables with 963 ICD-9 diseases and 424 ICD-9 disease families as dependent variables or endpoints.

### Model training and evaluation

To evaluate an indication prediction method for multiple drugs to multiple diseases, there are three possible approaches- (1) Global metrics: one can merge the prediction scores for all drugs over all diseases, and then compute the overall evaluation result; (2) Drug-centric metrics: one can compute an evaluation result for each drug and then average the results over all drugs to obtain an overall score; (3) Disease-centric metrics: one can compute an evaluation result for each disease and then average the results over all diseases to obtain an overall score. In this study, global metrics were used during the model training and cross-validation. All three evaluation approaches were implemented during the independent validation.

**Figure 2:**
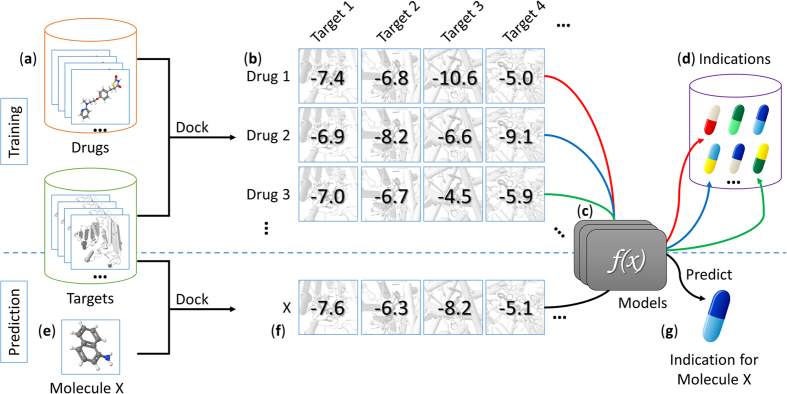
Flow chart of the model training and prediction process. We collected 1,256 drug molecules and 611 ligand-bindable targets (**a**) to constructed an *in silico* chemical-protein interactome (CPI) using docking (**b**). Based on the existing drug-indication knowledge, machine learning models (**c**) were trained to predict drug indications (**d**) based on the CPI. When a user submits a molecule to our server (**e**), it is docked against our library targets to generate docking scores. These scores are fed to the machine learning models (**f**) to predict the indications (**g**) for this molecule.

The workflow of the model training and prediction is shown in Fig. 2. We randomly split the original dataset into two equal parts, one half serving as training set, and the other half as independent validation set. We filtered the diseases that have fewer than five associated drugs in the new training set to ensure each endpoint has at least five positive samples. After the filtering process, we ended up having 638 ICD-9 individual diseases and 328 disease families. We treated the indication prediction task as a binary classification problem and constructed separate classifiers for each disease. A comparison of Naïve Bayes, logistic regression and random forest models showed comparable efficiency and accuracy of predictions on our training data, so we chose logistic regression for the DPDR-CPI server. The models were set up with L2-regularization which gives an increasing penalty as model complexity increases to prevent overfitting. Models were constructed using Python 2.7 and the Scikit-Learn package^51^ and evaluated with 10-fold cross-validation. Cross-validation experiments were repeated 100 times to get a mean and a standard deviation of the AUROCs and the AUPRs and the accuracy, precision, sensitivity, and specificity measures were calculated based on a prediction threshold when the maximum F-score (harmonic mean of precision and recall) was achieved.

Then we assessed the models on the independent validation data by using global metrics, drug-centric metrics, and disease-centric metrics. Since this independent dataset was not included anywhere in the training, we used it as a gold standard to evaluate our method. To compare our method against structural descriptor-based methods, we generated the E-state, ECFP6, FCFP6, FP4, Klekota-Roth, MACCS and PubChem^33^ fingerprints for all the drugs. The E-State, ECFP6, MACCS, and PubChem fingerprints were generated using rcdk package 3.3.2^52^ in R 3.1.3, FP4 fingerprints were produced by Open Babel 2.3.2^53^ and FCFP6 and Klekota-Roth fingerprints were generated via RDKit version 2016-06-30 in Anaconda Python 2.7.12. We built models based on the descriptor features following the same procedure above and compared the methods during the independent validation.

We also utilized all the data, including both the training and validation sets, to train comprehensive models to run on the server-side for predictions. The parameters and thresholds were determined using the exact cross-validation procedure described above. In order to make the scores comparable across different diseases for ranking purposes, we used an Empirical Bayes method^54^ to normalize prediction scores of the same drug across all endpoints (i.e., diseases). To explain this process, consider a particular drug *i* and divide the diseases into two groups: group 1 includes diseases which can be treated by drug *i*, and group 0 includes diseases which cannot be treated by drug *i*. For a disease *j*, *y*_*j*_ is the predicted score generated from the models. We use the confidence of disease *j* belonging to Group 1 (i.e. the probability of the disease belongs to Group 1 based on all predicted scores for drug *i*) as the normalized value.

According to the Bayes’s rule,


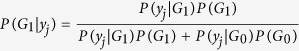


Here *P*(·) denotes the probability of an event. *G*_*1*_ and *G*_*0*_ denote the events of belonging to Group 1 and Group 0, respectively, and *y*_*j*_|*G*_*1*_ denotes the event of observing *y*_*j*_ when the disease belongs to Group 1. We obtain the probabilities on the right-hand side of the formula from empirical distributions. *P(G*_*0*_) and *P(G*_*1*_) are the prior probabilities of a disease from Group 0 and Group 1, respectively. *P(G*_*0*_) is the proportion of diseases that cannot be treated by the drug from the training data, and *P(G*_*1*_) is the proportion of diseases that can be treated by the drug from the training data. Let *P(y*_*j*_|*G*_*0*_) denotes the probability density from the distribution of predicted scores of diseases from Group 0 based on the training data. *P(y*_*j*_|*G*_*1*_) is the probability density from the distribution of predicted scores of diseases from Group 1 based on the training data. After obtaining all values on the right-hand side of the formula, the normalized score is calculated. Since the probabilities on the right-hand side are obtained from for each drug, the normalized scores of diseases are comparable within each drug.

### Server workflow

**Figure 3:**
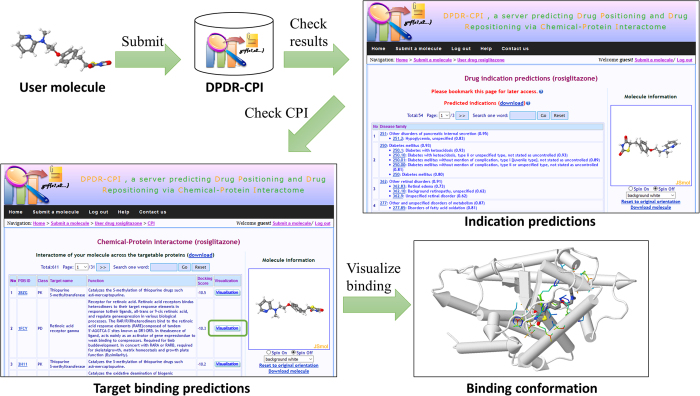
Workflow of the server. The user can submit a molecule in the format of MOL, MOL2, PDB, SDF or SMILES to the DPDR-CPI server. After the calculation is finished, the server will provide the indication predictions with probability values grouped by ICD-9 disease family. Then the user can check the target binding scores of the molecule across our 611 library targets. By clicking on the “Visualization” button, the user is able to view the interactive 3D binding confirmation between the molecule and any specific target.

The overall workflow of the server is shown in Fig. 3. Users can submit a molecular file in the following formats: MOL, MOL2, PDB, SDF and SMILES. A JSME Molecule Editor^55^ is also provided for the user to sketch a molecule. We utilize Molconvert 14.8.18.0 from Marvin Beans (https://www.chemaxon.com) and AutoDock Tools 1.5.4^56^ to convert the 2D molecular structure to 3D PDBQT file with Gasteiger charges. A small molecule, naphthylamine, is provided for a quick test of the server. Our server is designed to dock small drug-like molecules so it may fail or generate inaccurate results for molecules that are larger than 900 Daltons, such as peptides and natural products, or small inorganic molecules that do not contain any rotatable bonds. When the molecule file is submitted, it is added to the queue to be docked by AutoDock Vina^32^ against the 611 targets with default parameters. The docking scores and poses with the lowest energy scores are extracted and sent to the machine learning models for indication prediction. A typical calculation task usually takes minutes to hours, depending on how complicated the input molecule is. The user can choose to view the ongoing process online as it executes, bookmark the task link and return later, or leave an email address and wait for a notice.

The following results will be provided when a task is complete:

1. The predicted indications from 963 ICD-9 indications of 424 ICD-9 disease families along with confidence values. The indication table is organized as a tree-like structure based on ICD-9 code hierarchy and ranked by the ICD-9 family confidence values.
2. The binding scores and structures of the user molecule towards the 611 library targets. The interaction patterns can be visualized online via JSMol (http://www.jmol.org) and the target residues within 6.4 Å distance^23^ from the ligand are highlighted.

### Disclaimer

This server is only for research purposes and the authors and their organizations are excluded from all liability for any costs, claims, expenses, charges, losses, damages or penalties of any kind incurred directly or indirectly arising from the use of this server.

## Additional Information

**How to cite this article**: Luo, H. *et al.* DPDR-CPI, a server that predicts Drug Positioning and Drug Repositioning via Chemical-Protein Interactome. *Sci. Rep.* **6**, 35996; doi: 10.1038/srep35996 (2016).

**Publisher’s note**: Springer Nature remains neutral with regard to jurisdictional claims in published maps and institutional affiliations.

## Supplementary Material

Supplementary Information

Supplementary Tables

## Figures and Tables

**Table 1:** Performance evaluation of DPDR-CPI using the entire dataset versus the training set during 10-fold cross-validations.

Endpoints	Dataset	Accuracy	Precision	Sensitivity	Specificity	AUROC	AUPR
638 ICD-9 diseases	Entire dataset	0.956 ± 0.000	0.176 ± 0.001	0.274 ± 0.002	0.972 ± 0.000	0.782 ± 0.001	0.151 ± 0.001
	Training set	0.953 ± 0.000	0.152 ± 0.001	0.241 ± 0.001	0.969 ± 0.000	0.752 ± 0.001	0.123 ± 0.001
328 ICD-9 disease families	Entire dataset	0.925 ± 0.000	0.167 ± 0.000	0.363 ± 0.001	0.942 ± 0.000	0.783 ± 0.000	0.169 ± 0.001
	Training set	0.919 ± 0.000	0.152 ± 0.001	0.341 ± 0.003	0.938 ± 0.000	0.760 ± 0.001	0.149 ± 0.001

The entire dataset was utilized to build server-side prediction models while the training set was used to construct models for independent validation. The training set is a half of the entire dataset.

**Table 2:** Performance comparisons of the different structural descriptor-based methods and DPDR-CPI using 638 endpoints of ICD-9 disease indications on the independent validation data.

Metric	Method	Accuracy	Precision	Sensitivity	Specificity	AUROC	AUPR
global	LR-ECFP6	0.801	0.040	0.355	0.811	0.651	0.040
	LR-E-State	0.792	0.033	0.305	0.802	0.602	0.030
	LR-FCFP6	0.904	0.050	0.193	0.919	0.670	0.051
	LR-FP4	0.870	0.045	0.247	0.883	0.633	0.038
	LR-KR	0.897	0.045	0.190	0.912	0.660	0.048
	LR-MACCS	0.881	0.047	0.235	0.896	0.649	0.039
	LR-PubChem	0.866	0.054	0.318	0.878	0.704	0.050
	DPDR-CPI	0.964	0.192	0.203	0.981	0.764	0.118
drug-centric (628)	LR-ECFP6	0.906 ± 0.140	0.316 ± 0.303	0.490 ± 0.267	0.916 ± 0.144	0.783 ± 0.154	0.135 ± 0.161
	LR-E-State	0.888 ± 0.153	0.235 ± 0.245	0.465 ± 0.273	0.898 ± 0.158	0.744 ± 0.150	0.085 ± 0.087
	LR-FCFP6	0.902 ± 0.147	0.298 ± 0.292	0.497 ± 0.267	0.911 ± 0.150	0.781 ± 0.153	0.132 ± 0.156
	LR-FP4	0.897 ± 0.145	0.258 ± 0.261	0.472 ± 0.266	0.907 ± 0.149	0.761 ± 0.146	0.103 ± 0.116
	LR-KR	0.903 ± 0.139	0.289 ± 0.290	0.498 ± 0.265	0.912 ± 0.142	0.778 ± 0.152	0.129 ± 0.156
	LR-MACCS	0.895 ± 0.148	0.271 ± 0.276	0.478 ± 0.269	0.905 ± 0.152	0.762 ± 0.152	0.108 ± 0.124
	LR-PubChem	0.897 ± 0.156	0.293 ± 0.294	0.486 ± 0.265	0.907 ± 0.160	0.766 ± 0.160	0.125 ± 0.146
	DPDR-CPI	0.893 ± 0.150	0.273 ± 0.282	0.511 ± 0.271	0.902 ± 0.154	0.775 ± 0.156	0.128 ± 0.163
disease-centric (638)	LR-ECFP6	0.668 ± 0.268	0.061 ± 0.075	0.559 ± 0.305	0.671 ± 0.278	0.563 ± 0.138	0.032 ± 0.036
	LR-E-State	0.596 ± 0.323	0.059 ± 0.097	0.593 ± 0.338	0.596 ± 0.336	0.504 ± 0.150	0.026 ± 0.026
	LR-FCFP6	0.711 ± 0.242	0.077 ± 0.105	0.529 ± 0.293	0.716 ± 0.250	0.583 ± 0.136	0.036 ± 0.044
	LR-FP4	0.629 ± 0.307	0.064 ± 0.093	0.575 ± 0.326	0.632 ± 0.318	0.524 ± 0.148	0.030 ± 0.035
	LR-KR	0.690 ± 0.259	0.109 ± 0.212	0.553 ± 0.308	0.695 ± 0.268	0.571 ± 0.133	0.035 ± 0.048
	LR-MACCS	0.659 ± 0.286	0.061 ± 0.083	0.549 ± 0.310	0.663 ± 0.296	0.536 ± 0.145	0.030 ± 0.034
	LR-PubChem	0.746 ± 0.231	0.075 ± 0.090	0.523 ± 0.283	0.752 ± 0.239	0.609 ± 0.144	0.039 ± 0.046
	DPDR-CPI	0.888 ± 0.173	0.258 ± 0.261	0.388 ± 0.234	0.899 ± 0.179	0.682 ± 0.148	0.088 ± 0.091

Three types of metrics, including global, drug-centric and disease-centric metrics, were used.

**Table 3:** Drug candidate positioning prediction for NM-702 using the DPDR-CPI server.

Rank	Disease	Confidence
1	458: Hypotension	0.80
	458: Hypotension	0.80
	458.9: Hypotension, unspecified	0.80
2	434: Occlusion of cerebral arteries	0.70
	434.91: Cerebral artery occlusion, unspecified with cerebral infarction	0.70
3	443: Other peripheral vascular disease	0.69
	443.9: Peripheral vascular disease, unspecified	0.69
4	427: Cardiac dysrhythmias	0.67
	427: Cardiac dysrhythmias	0.60
	427.1: Paroxysmal ventricular tachycardia	0.59
	427.9: Cardiac dysrhythmia, unspecified	0.58

The diseases are grouped into ICD-9 families and ranked by their confidence values.

**Table 4:** Top disease predictions for rosiglitazone from the server.

Rank	Disease	Confidence
1	251: Other disorders of pancreatic internal secretion	0.95
	251.2: Hypoglycemia, unspecified	0.83
2	250: Diabetes mellitus	0.93
	250.1: Diabetes with ketoacidosis	0.93
	250.10: Diabetes with ketoacidosis, type ii or unspecified type, not stated as uncontrolled	0.93
	250.01: Diabetes mellitus without mention of complication, type i [juvenile type], not stated as uncontrolled	0.89
	250.00: Diabetes mellitus without mention of complication, type ii or unspecified type, not stated as uncontrolled	0.81
	250: Diabetes mellitus	0.80
3	362: Other retinal disorders	0.91
	362.83: Retinal edema	0.73
	362.10: Background retinopathy, unspecified	0.62
	362.9: Unspecified retinal disorder	0.62
4	277: Other and unspecified disorders of metabolism	0.87
	277.85: Disorders of fatty acid oxidation	0.81
5	276: Disorders of fluid electrolyte and acid-base balance	0.87
	276.2: Acidosis	0.87
	276.69: Other fluid overload	0.64
6	365: Glaucoma	0.85
	365: Glaucoma	0.85
	365.9: Unspecified glaucoma	0.85
	365.1: Open-angle glaucoma	0.84
	365.10: Open-angle glaucoma, unspecified	0.84
	365.13: Pigmentary open-angle glaucoma	0.84
	365.04: Ocular hypertension	0.84
	365.00: Preglaucoma, unspecified	0.82
7	331: Other cerebral degenerations	0.83
	331.0: Alzheimer’s disease	0.82

The diseases are grouped into ICD-9 families and ranked by their confidence values.
