# Supplementary material for: DPDR-CPI, a server that predicts Drug Positioning and Drug Repositioning via Chemical-Protein Interactome
Source: Sci Rep. 2016 Nov 2;6:35996. doi: 10.1038/srep35996 (PMC5090963; doi:10.1038/srep35996)
Supplement: Supplementary Information [file srep35996-s1.doc]

**DPDR-CPI, a server that predicts Drug Positioning and Drug Repositioning via Chemical-Protein Interactome**

Heng Luo^#,1,2^, Ping Zhang^#,2^, Xi Hang Cao^3^, Dizheng Du^1^, Hao Ye^1^, Hui Huang^1^, Can Li^1^, Shengying Qin^1^, Chunling Wan^1^, Leming Shi^4^, Lin He^1^, Lun Yang*^,1,$^

^1^Bio-X Institutes, Shanghai Jiao Tong University, Shanghai 200030, China

^2^Center for Computational Health, IBM T.J. Watson Research Center, Yorktown Heights, NY 10598, USA

^3^Center for Data Analytics and Biomedical Informatics, Temple University, Philadelphia, PA 19122, USA

^4^School of Pharmacy, Fudan University, Shanghai 201203, China

**
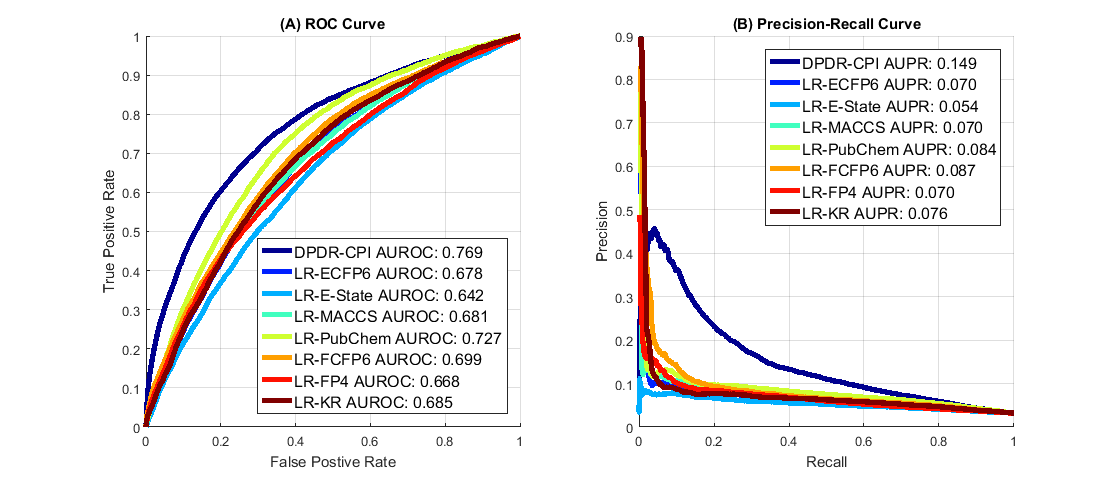
**

**Supplementary Figure 1.** Under global metric, (A) the ROC curve comparison and (B) the precision-recall curve comparison for different prediction methods of 328 ICD-9 disease families on the independent validation data
